# Enhancing predictions of health insurance overspending risk through hospital departmental performance indicators

**DOI:** 10.17305/bb.2025.12051

**Published:** 2025-06-28

**Authors:** Yao Bu, Danqi Wang, Xiaomao Fan, Jiongying Li, Lei Hua, Lin Zhang, Wenjun Ma, Liwen He, Hao Zang, Haijun Zhang, Xingyu Liu, Yufeng Gao, Li Liu

**Affiliations:** 1 School of Artificial Intelligence and Computer Science, Jiangnan University, Wuxi, Jiangsu, China; 2 Big Data Center, Affiliated Hospital of Jiangnan University, Wuxi, Jiangsu, China; 3 College of Big Data and Internet, Shenzhen Technology University, Shenzhen, Guangdong, China; 4 Office of Health Insurance Administration, Affiliated Hospital of Jiangnan University, Wuxi, Jiangsu, China; 5 Suzhou Industrial Park Monash Research Institute of Science and Technology, Monash University, Suzhou, Jiangsu, China; 6 Monash University-Southeast University Joint Research Institute (Suzhou), Southeast University, Suzhou, Jiangsu, China; 7 School of Computer Science, South China Normal University, Guangzhou, Guangdong, China; 8 Wuxi Innovation Center, Shenzhen Research Institute of Big Data, Wuxi, Jiangsu, China; 9 School of Information and Control Engineering China University of Mining and Technology, Xuzhou, Jiangsu, China; 10 Jiangsu Zhisheng Information Technology Co., LTD., Xuzhou, Jiangsu, China; 11 Wuxi Health Statistics and Information Center, Wuxi, Jiangsu, China; 12 Affiliated Hospital of Jiangnan University, Wuxi, Jiangsu, China

**Keywords:** Health insurance overspending, departmental performance indicators, DPIs, overspending risk prediction, machine learning, health insurance management system, HIMS

## Abstract

The substantial rise in health insurance expenditures, combined with delayed feedback on overspending from administrative departments, highlights the urgent need for timely reporting of such data. This study analyzed a large cohort of 549,910 discharged patients’ medical records from the Wuxi Health Commission, covering the period from January 2022 to November 2023. We applied four widely recognized machine learning techniques—logistic regression (LR), LightGBM, random forest (RF), and artificial neural networks (ANNs)—alongside departmental performance indicators (DPIs) to develop insurance overspending risk prediction (IORP) models at both regional and hospital levels. The dataset was divided into training and testing sets in a 7:3 ratio. Experimental results showed that LightGBM outperformed the other models, achieving an accuracy of 0.82 for both regional and hospital-level predictions. Its weighted F1-score reached 0.78 at the regional level and 0.82 at the hospital level, with corresponding area under the receiver operating characteristic curve (AUC-ROC) values of 0.91 and 0.94, demonstrating strong performance in identifying overspending risks. The model’s high recall and precision further ensure reliable predictions and minimize misclassifications. Notably, four key DPIs—total amount of discharged patients (TADPs), average inpatient stay (AIS), medicine expenses percentage (MEP), and consumable expenses percentage (CEP)—were strongly correlated with overspending risks. The integration of IORP models into the Health Insurance Management System (HIMS) at the Affiliated Hospital of Jiangnan University has significantly improved departmental managers’ ability to anticipate overspending. By effectively leveraging HIMS in combination with this advanced model, managers can perform timely, accurate assessments, thereby enhancing financial oversight and resource allocation.

## Introduction

The escalating costs of healthcare have become a global concern, with overspending posing significant challenges to the financial sustainability of healthcare systems. In many countries, healthcare expenditures have grown at an unsustainable rate, driven by factors such as aging populations, the increasing prevalence of chronic diseases, and the rising costs of medical technologies and pharmaceuticals [1, 2]. Overspending in healthcare not only strains national budgets but also threatens the equitable allocation of resources, potentially compromising the quality of care and access to essential services [3]. For instance, in China, the rapid expansion of national health insurance coverage has led to increased financial pressures on hospitals, with overspending becoming a critical issue that undermines the efficiency of healthcare delivery [4]. However, we found that delayed feedback on overspending—an issue never before addressed internationally—has significantly hindered hospital departmental managers’ ability to make timely, informed adjustments. Therefore, the development of a system for predicting overspending risks is crucial, enabling administrators to make prompt decisions and enhance the management of health insurance expenditures at both regional and hospital levels. Recently, researchers have investigated various causes of high medical insurance costs and proposed a series of corresponding evaluation and prediction approaches [5–15]. These approaches can be categorized into three groups: statistical analysis [5, 10–14], machine learning modeling [6–9, 14], and deep learning methods [6, 15]. Regarding statistical analysis-based methods, Mitkova et al. [10] proposed using the Kruskal–Wallis test to analyze current and extrapolate future trends in healthcare and pharmaceutical budgets based on the National Health Insurance Fund (NHIF). Murakami et al. [11] used Gamma regression to analyze data from 33,213 cardiovascular disease patients, aiming to identify risk factors correlated with medical expenses and reduce overall healthcare costs. Based on data primarily from 2013–2016, Papanicolas et al. [12] analyzed information from key international organizations in the Organisation for Economic Co-operation and Development (OECD) and found that services, drug expenses, medical management costs, and employee salaries are critical factors contributing to the high costs incurred by hospitals. Regarding machine learning-based methods, Ye [14] selected population factors as independent variables and urban basic medical insurance expenditure as the dependent variable, establishing a regression model to explore their relationships. Using inpatient data from the National Health Research Database (NHRD), Huang et al. [7] constructed a predictive model employing various machine learning algorithms, including support vector regression (SVR) and extreme gradient boosting (XGBoost), and found that surgical expenses were a major cost factor for patients. Kaushik et al. [8] predicted individual health insurance costs based on demographic features and achieved an accuracy of 92.72%. Additionally, several studies have integrated machine learning methods into healthcare information systems to enhance predictive capabilities and decision support [16–20]. These works provide insights into implementation strategies that reinforce the novelty of our HIMS integration. Regarding deep learning-based methods, Zhang et al. [15] proposed a framework for detecting fraud in medical insurance using consortium blockchain technology and deep learning, which improved efficiency and effectively identified fraud. Drewe-Boss et al. [6] used a deep neural network and a ridge regression model on a sample of German insurants to predict total one-year healthcare costs, finding that the neural network demonstrated superior performance. While these methods achieved competitive predictive performance, they primarily focused on controlling individual medical expenses. As such, they show limited effectiveness in addressing overspending at regional and hospital levels, while also imposing heavy administrative burdens on managers overseeing departmental spending. From a regional and hospital perspective, departmental decision-making plays a more crucial role in managing costs. Strengthening budget management at the departmental level allows hospitals to more effectively control expenditures while maintaining a balance between the quality of medical services and financial stability. To address the aforementioned issues, we propose the Health insurance overspending risk prediction (IORP) models using departmental performance indicators (DPIs) for regional and hospital administrators. Specifically, we first collected 549,910 discharged patient medical records from January 2022 to November 2023 in Wuxi, China. These records were aggregated into regional-level and hospital-level departmental datasets, containing 8,416 and 44,017 records, respectively. In addition, we employed statistical process control (SPC) techniques to categorize departmental overspending into three groups: high risk, low risk, and no risk.

Next, we utilized four widely recognized machine learning techniques—logistic regression (LR), LightGBM, random forest (RF), and artificial neural networks (ANNs)—with DPIs to develop regional- and hospital-level IORP models. The experimental results show that the LightGBM algorithm exhibited outstanding predictive capabilities, achieving an accuracy of 0.82 for both regional- and hospital-level models. We then used SHapley Additive exPlanations (SHAP) to present the importance of each DPI. Our analysis identified four key indicators strongly correlated with departmental overspending: total amount of discharged patients (TADPs), average inpatient stay (AIS), medicine expenses percentage (MEP), and consumable expenses percentage (CEP). Finally, we integrated the IORP models into the Hospital Information Management System at the Affiliated Hospital of Jiangnan University to enhance administrators’ ability to predict overspending risks. By effectively utilizing this advanced model within HIMS, hospital departmental managers can conduct timely and accurate risk assessments, leading to more efficient financial management and optimal resource allocation. To summarize, the primary contributions of this study are as follows:

- We collected 549,910 discharged patient records from January 2022 to November 2023 in Wuxi, China, and organized them into both regional- and hospital-level departmental datasets. We applied SPC techniques to categorize departmental overspending into three distinct risk groups: no risk, low risk, and high risk.
- We employed four widely recognized machine learning techniques—LR, LightGBM, RF, and ANN—using DPIs to develop regional- and hospital-level IORP models.
- We identified four key indicators strongly correlated with departmental overspending: TADP, AIS, MEP, and CEP.
- We successfully integrated the IORP models into the Hospital Information Management System at the Affiliated Hospital of Jiangnan University. This integration facilitates timely and accurate risk assessments, significantly improving financial management and resource allocation.

The remainder of this paper is organized as follows. Section 1 provides a detailed overview of the data and methods. Section 2 discusses the results, including model evaluation, explanation, and applications. Section 3 explores the principal findings and limitations. Finally, Section 4 concludes the paper.

## Materials and methods

### Study design

**Figure 1.**
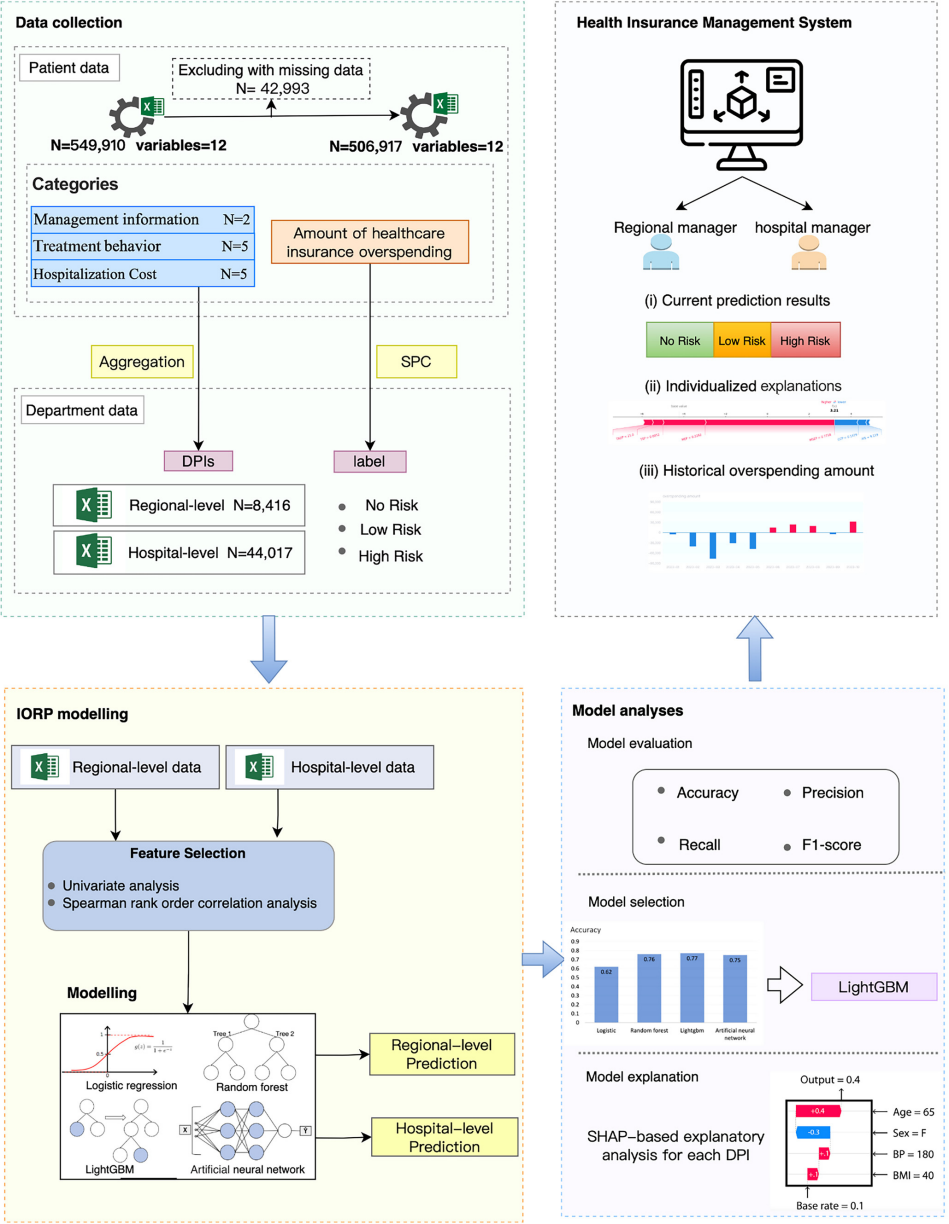
**The overview of IORP model and analyses**. IORP: Insurance overspending risk prediction; SHAP: SHapley Additive exPlanations; DPI: Departmental performance indicator; SPC: Statistical process control.

This study utilized a local health insurance database containing medical records of discharged patients in China. Our methodology began with the extraction of DPIs from individual patient insurance data to forecast departmental overspending risks. We then constructed two datasets: one for monthly departmental data at the regional level and another for daily departmental data at the hospital level. To classify overspending (i.e., the label) across various departments, we employed SPC, a widely recognized method for quality assurance in industrial settings. For IORP modeling at both regional and hospital levels, we applied four machine learning algorithms: LR, RF, LightGBM, and ANNs. The most effective models were selected to predict hospital overspending with high accuracy. Additionally, we utilized SHAP to interpret and visualize the contribution of each DPI to the target risk status. To support regional and hospital administrators, we developed a Hospital Information Management System that facilitates monitoring of health insurance overspending and enables timely adjustments. An overview of our IORP framework is illustrated in Figure 1.

### Data collection and preprocessing

**Figure 2.**
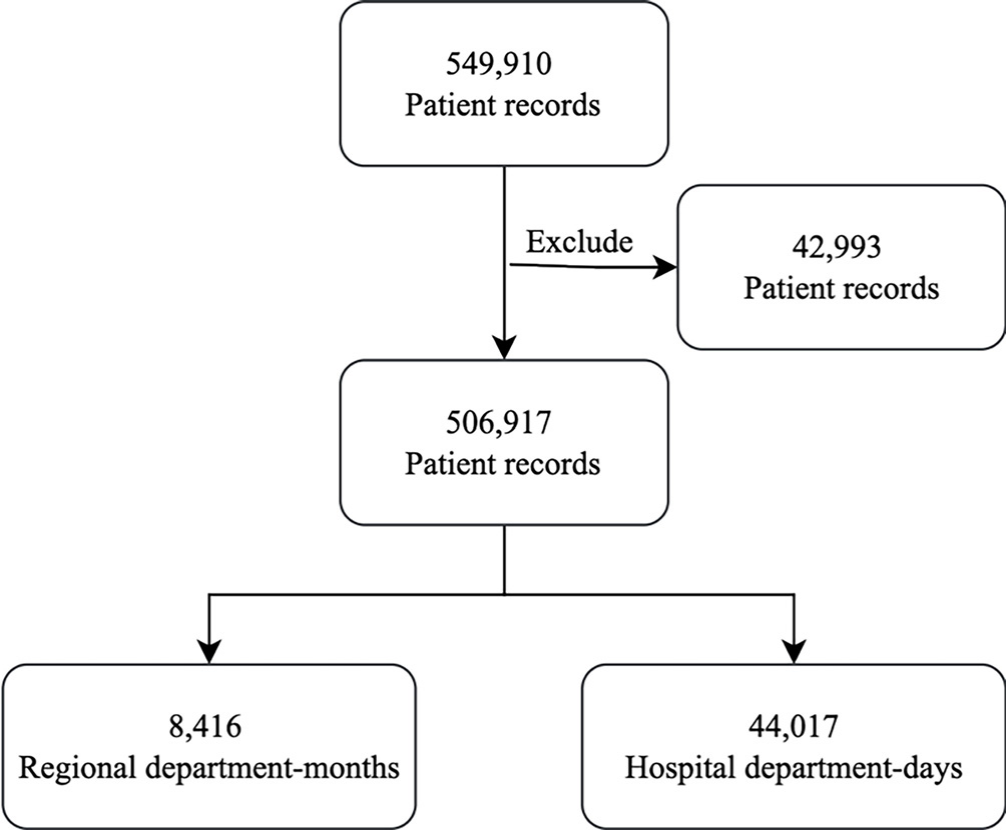
**Data processing flowchart for patient record inclusion and department-level aggregation**.

We obtained medical records of discharged patients, complete with health insurance information (*N* ═ 549,910), from the Wuxi Health Commission. These records cover the period from January 2022 to November 2023. We included a total of 12 variables, categorized into three groups: management information (*n* ═ 2), treatment behavior (*n* ═ 5), and hospitalization costs (*n* ═ 5), as detailed in Table S3. The dataset had a 7.82% missing data rate, with many missing values related to consumable costs. Since consumable costs can vary greatly between departments and the overall impact was limited due to the small missing ratio, we decided to exclude 42,993 patient records with missing features to maintain data integrity (see Figure 2). After this exclusion, we retained 506,917 samples for analysis. We aggregated the discharged patient records to create department-level datasets featuring 8 DPIs. The regional datasets were compiled on a monthly basis (*N* ═ 8,416) to assist regional administrators in tracking the overspending risk status of departments. For hospital-level overspending risk predictions, the dataset was generated cumulatively on a daily basis (*N* ═ 44,017). Among the 8 DPIs, 5 pertained to treatment behavior and 3 to hospitalization costs (see Table 1).

**Table 1:** The description of DPIs for departmental datasets

**Category**	**DPIs**	**Description**
Treatment behavior	Total amount of discharged patients (TADP)	Total amount of discharged patients
	Critical cases percentage (CCP)	Proportion of critical patients to the total amount of discharged patients
	Total surgery percentage (TSP)	Proportion of discharged patients undergoing surgeries to the total amount of discharged patients
	IV-surgery percentage (IVSP)	Proportion of discharged patients undergoing IV-surgeries to discharged patients undergoing surgeries
	Average inpatient stay (AIS)	Average length of inpatient stay
Hospitalization costs	Medicine expenses percentage (MEP)	Proportion of medicine expenses to total expenses
	Consumables expenses percentage (CEP)	Proportion of consumables expenses to total expenses
	Medical service expenses percentage (MSEP)	The deduction of total cumulative expenses to medicine and consumables expenses

DPIs: Departmental performance indicators.

**Table 2:** The hyperparameter tuning range of different algorithms and the optimal hyperparameter combination for each algorithm

**Algorithm**	**Range of hyperparameters**	**Regional modeling hyperparameters**	**Hospital modeling hyperparameters**
LR	C: (1e-5,100); max_iter: (100, 1000); solver: {‘liblinear’, ‘lbfgs’, ‘newton-cg’, ‘sag’, ‘saga’}	‘C’: 33.37; ‘max_iter’: 872; ‘solver’: ‘liblinear’	/
RF	n_estimators: (20,200); max_depth: (2,256); min_samples_leaf: (1,64); max_samples: (0.5,1.0); criterion: {‘gini’, ‘entropy’}; random_state: (1,100)	‘n_estimators’: 181; ‘max_depth’: 92; ‘min_samples_leaf’: 1; ‘max_samples’: 0.9; ‘criterion’: ‘entropy’; ‘random_state’: 70	‘n_estimators’: 75; ‘max_depth’: 150; ‘min_samples_leaf’: 1; ‘max_samples’: 0.85; ‘criterion’: ‘gini’; ‘random_state’: 14
LightGBM	n_estimators: (20,200); max_depth: (2,256); learning_rate(0.01,0.2); min_child_samples(5,100)	‘n_estimators’: 30; ‘max_depth’: 254; ‘learning_rate’: 0.1; ‘min_child_samples’: 10	‘n_estimators’: 200; ‘max_depth’: 145; ‘learning_rate’: 0.19; ‘min_child_samples’: 45
ANN	layers: (1,3); units_per_layer: (32,512); activation {‘relu’, ‘tanh’, ‘sigmoid’}	‘layers’: 2; ‘units_per_layer’: 436; ‘activation’: ‘relu’	/

LR: Logistic regression; RF: Random forest; ANN: Artificial neural network.

We categorized departments into 7 groups: tumors, burns, general medicine, integrated sections, surgery, obstetrics and gynecology, and severe illnesses. To assess overspending risk within each group, we utilized SPC to define the following risk levels:

- No Risk: An overspending amount less than zero indicated the department was within budget and operating at a surplus; these were uniformly classified as no risk.
- Low Risk: Overspending below the centerline was classified as low risk, indicating that while the budget was exceeded, the deviation remained within acceptable limits.
- High Risk: For departments with overspending above zero, the centerline (mean) was calculated. Any overspending above this centerline was classified as high risk, indicating significant deviation from expected expenditure patterns that warrants immediate attention and corrective action.

### Statistical methods

A descriptive analysis of DPIs is presented in Table 1, with all variables summarized using mean and standard deviation (SD). Student’s *t*-tests were used to compare groups (no risk vs low risk; low risk vs high risk; no risk vs high risk). Statistical tests were performed using the Python scipy package (v1.7.3). Normality was assessed using the Shapiro–Wilk test (all *P* > 0.05), and homogeneity of variance using Levene’s test (all *P* > 0.05). Full results are provided in Table S4.

Univariate analysis was conducted on the training set to evaluate the association between each DPI and the target overspending risks (Python sklearn package (v1.0.2)), considering the DPI with a *P* value less than 0.05 to have a significant difference with the label and thus included in modeling. Subsequently, we performed a pairwise Spearman’s rank order correlation analysis (Python scipy package (v1.7.3)) on all DPIs. Redundancy was examined for features with coefficients greater than 0.70. Expert opinion and prediction effectiveness were taken into account when selecting DPIs for IORP modeling.

### IORP modelling

We randomly divided the full dataset into training and test sets (70:30), followed by data scaling using MinMaxScaler (Python sklearn package v1.0.2). For overspending modeling, we selected four machine learning algorithms: LR, RF, LightGBM, and ANN. LR maps feature combinations to probabilities using a sigmoid function for classification [21]. RF enhances generalization by aggregating multiple decision trees [22]. LightGBM optimizes gradient boosting decision trees (GBDTs) using techniques like histogram-based learning and feature bundling for efficiency. ANN, inspired by biological neurons, learns complex patterns through layered computations [23]. We used 5-fold cross-validation on the training set. In each iteration, 80% of the data were used for training and 20% for validation. During cross-validation, hyperparameters were optimized using the Optuna framework (tree-structured Parzen estimator, TPE) (Python optuna package v3.0.4), with the objective of maximizing model accuracy. For the RF model, the number of trees (n_estimators) was optimized, with optimal values of 181 for regional modeling and 75 for hospital modeling. For the LightGBM model, we focused on max_depth, which controls tree depth, with optimal values of 254 (regional) and 145 (hospital). Since class imbalance was moderate, no class weighting or resampling techniques were applied. Tree-based models like LightGBM are generally robust to moderate imbalance. Per-class metrics were used to monitor performance. The hyperparameter tuning ranges and optimal values for all algorithms are summarized in Table 2.

### Ethical statement

The study received approval from the Medical Ethics Committee Review Board of the Affiliated Hospital of Jiangnan University in 2022 (Approval No. LS2022110). Informed consent was deemed unnecessary.

### Statistical analysis

We employed multiple performance metrics to assess the predictive performance of the classification models: accuracy [24], recall, precision, and F1-score [25], using the Python sklearn package (v1.0.2). The formulas used are summarized in Table S1.

To assess feature contributions, we used the SHAP algorithm [26, 27], which provided global and individual-level interpretations of each DPI’s importance in predicting overspending risk. In our analysis of the best-performing model, four DPIs showed strong correlation with high-risk overspending: TADPs, AIS, MEP, and CEP. Individualized feature importance plots were generated using the test dataset. All analyses and visualizations were performed using Python scikit-learn (v1.0.2) and shap (v0.41.0).

## Results

### The characteristics of the study population and departmental datasets

The 12 variables from discharged patients’ medical records related to overspending prediction are presented in Table S2. They mainly comprised management information (16.7%), treatment behavior (41.7%), and hospitalization costs (41.7%). We removed 42,993 patients (7.82%) with missing data. Comparisons between different risk factor categories were conducted using the Student’s *t*-test (significance level of 0.05). Numerical variables were presented as mean (SD), and categorical variables as number (percentage) (Table 3).

**Table 3:** Baseline characterization of departmental data

**DPIs**	**Mean (SD)**				***P* value**		
	**Overall**	**High risk**	**Low risk**	**No risk**	**High vs Low**	**High vs No**	**Low vs No**
*Region*							
TADP	81.87 (77.96)	92.68 (85.97)	60.01 (59.43)	86.14 (69.49)	<0.001	0.02	<0.001
CCP	0.38 (0.33)	0.35 (0.31)	0.4 (0.36)	0.44 (0.35)	<0.001	<0.001	<0.001
TSP	0.78 (0.29)	0.77 (0.31)	0.77 (0.27)	0.81 (0.21)	<0.001	<0.001	<0.001
IVSP	0.14 (0.22)	0.13 (0.21)	0.16 (0.24)	0.17 (0.25)	<0.001	<0.001	0.15
MEP	0.23 (0.12)	0.22 (0.11)	0.23 (0.11)	0.25 (0.14)	<0.001	<0.001	<0.001
CEP	0.15 (0.14)	0.16 (0.14)	0.13 (0.13)	0.15 (0.16)	<0.001	0.01	<0.001
MSEP	0.77 (0.12)	0.78 (0.11)	0.77 (0.11)	0.75 (0.14)	<0.001	<0.001	<0.001
AIS	7.87 (7.5)	5.87 (3.3)	9.34 (6.81)	12.69 (14.68)	<0.001	<0.001	<0.001
*Hospital*							
TADP	54.38 (55.16)	62.4 (48.04)	33.66 (34.99)	61.52 (61.27)	<0.001	0.29	<0.001
CCP	0.29 (0.27)	0.37 (0.28)	0.35 (0.3)	0.25 (0.25)	<0.001	<0.001	<0.001
TSP	0.76 (0.26)	0.75 (0.22)	0.74 (0.26)	0.77 (0.27)	<0.001	<0.001	<0.001
MEP	0.26 (0.1)	0.28 (0.1)	0.27 (0.1)	0.26 (0.1)	<0.001	<0.001	<0.001
CEP	0.16 (0.13)	0.17 (0.13)	0.17 (0.13)	0.16 (0.13)	<0.001	<0.001	<0.001
MSEP	0.77 (0.12)	0.78 (0.11)	0.77 (0.11)	0.75 (0.14)	<0.001	<0.001	<0.001
AIS	7.87 (7.5)	5.87 (3.3)	9.34 (6.81)	12.69(14.8)	<0.001	<0.001	<0.001

TADP: Total amount of discharged patient; CCP: Critical cases percentage; TSP: Total surgery percentage; MEP: Medicine expenses percentage; CEP: Consumable expenses percentage; MSEP: Medical service expenses percentage; AIS: Average inpatient stay; IVSP: IV-surgery percentage.

### The selection of DPIs

According to the univariate analysis, all 8 DPIs were statistically significant, with all *P* values less than 0.001. As shown in Figure S1B, the hospital-level Spearman correlation analysis revealed that IVSP was highly correlated with CEP, with a correlation coefficient of 0.75. Since CEP directly reflects the proportion of hospital consumables expenditure, it is a key indicator for measuring overspending risk in many departments and plays an important role in total hospital expenditure. Therefore, we decided to exclude IVSP to help alleviate multicollinearity. As shown in Table S3, the Student’s *t*-test (*P* value > 0.05) between the training and test sets demonstrated the validity of modeling with the selected DPIs.

To further assess the robustness of the identified differences, we conducted a statistical power analysis for both regional- and hospital-level indicators. The results confirmed adequate power for all pairwise comparisons, supporting the validity of our findings. Detailed results are provided in Tables S5 and S6.

### Regional-level and hospital-level prediction results

The performance of different algorithms in predicting overspending status is shown in Table 4. At the regional level, LightGBM and RF outperformed other models, with accuracy, weighted precision, recall, and F1-score all above 0.70. The F1-scores of LightGBM and RF were 0.78 and 0.72, respectively. The accuracy, weighted precision, recall, and AUC-ROC (area under the ROC curve) of LightGBM were 0.82, 0.78, 0.78, and 0.91.

**Table 4:** Regional and hospital performance across models

**Algorithm**	**Accuracy**	**Weighted avg.**			
		**Precision**	**Recall**	**F1-score**	**AUC-ROC**
*Region*					
LR	0.63	0.63	0.63	0.56	0.71
RF	0.72	0.72	0.72	0.72	0.87
LightGBM	0.82	0.78	0.78	0.78	0.91
ANN	0.69	0.68	0.69	0.67	0.79
*Hospital*					
RF	0.74	0.74	0.74	0.74	0.88
LightGBM	0.82	0.82	0.82	0.82	0.94

LR: Logistic regression; RF: Random forest; ANN: Artificial neural network.

According to the evaluation results of the regional models, LR and ANN performed poorly, with accuracy, weighted precision, and recall all below 0.7. In particular, LR’s accuracy and weighted F1-score were 0.63 and 0.56, while ANN’s accuracy and weighted F1-score were 0.69 and 0.67. In contrast, RF and LightGBM showed higher accuracy and weighted F1-scores. Given these results, we excluded LR and ANN from the final hospital-level experiment, as they did not meet the performance thresholds required for reliable hospital-level overspending prediction. As shown in Table 4, LightGBM achieved an accuracy and weighted F1-score of 0.82, and a weighted AUC-ROC of 0.94. Meanwhile, RF achieved an accuracy, weighted precision, recall, and F1-score of 0.74, and a weighted AUC-ROC of 0.88.

**Figure 3.**
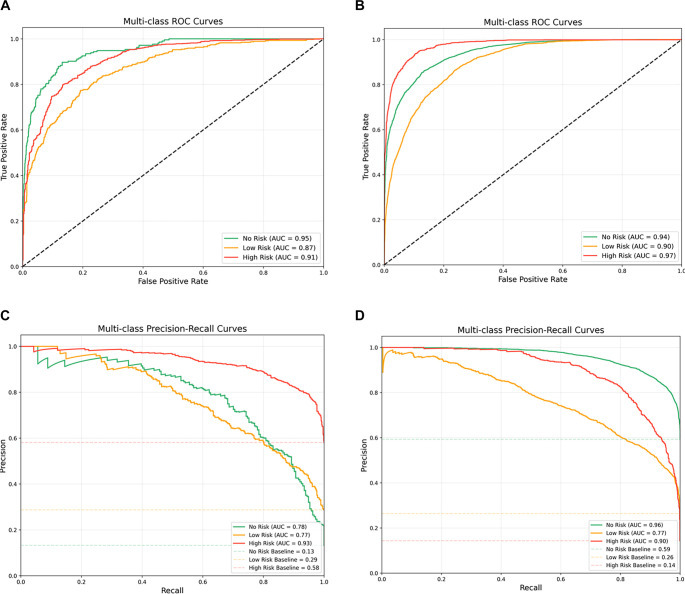
**ROC and PR curves of the LightGBM model for regional- and hospital-level predictions.** (A) Regional-level ROC curve; (B) Hospital-level ROC curve; (C) Regional-level PR curve; (D) Hospital-level PR curve. ROC: Receiver operating characteristic; PR: Precision–Recall.

For the LightGBM model, which performed the best, we report accuracy, precision, recall, F1-score, and AUC-ROC scores and curves (Figure 3A and 3B) for each classification (high risk, low risk, and no risk). As shown in Table 5, at the regional level, LightGBM achieved a high-risk classification accuracy of 0.85 and an AUC-ROC of 0.91. At the hospital level, the model maintained excellent performance for the high-risk class, with an accuracy of 0.82 and an AUC-ROC of 0.97. In addition, we present the PR-AUC curves for each classification of the LightGBM model at the regional and hospital levels in Figure 3C and 3D. At both regional and hospital levels, the high-risk class achieved PR-AUC values of 0.93 and 0.90, respectively, indicating a good precision–recall trade-off and excellent identification capability for high-risk departments. To further assess classification performance, confusion matrices are provided in Figure S2. Moreover, calibration analysis (Figure S3) showed Brier scores of 0.06 and 0.05 for the regional and hospital models, respectively. In both cases, the calibration curves closely followed the 45∘ diagonal, suggesting acceptable probability calibration.

**Table 5:** Prediction performance of the LightGBM model across region and hospital for each risk category

	**Accuracy**	**Precision**	**Recall**	**F1-score**	**AUC-ROC**
*Region*					
No risk	0.86	0.87	0.86	0.86	0.95
Low risk	0.73	0.73	0.73	0.73	0.87
High risk	0.85	0.81	0.85	0.83	0.91
*Hospital*					
No risk	0.90	0.87	0.90	0.89	0.94
Low risk	0.65	0.72	0.65	0.68	0.90
High risk	0.82	0.80	0.82	0.81	0.97

These results demonstrate that the LightGBM model can provide reliable predictions for high-risk cases, which is crucial for practical deployment in HIMS.

### Discoveries from IORP modeling

**Figure 4.**
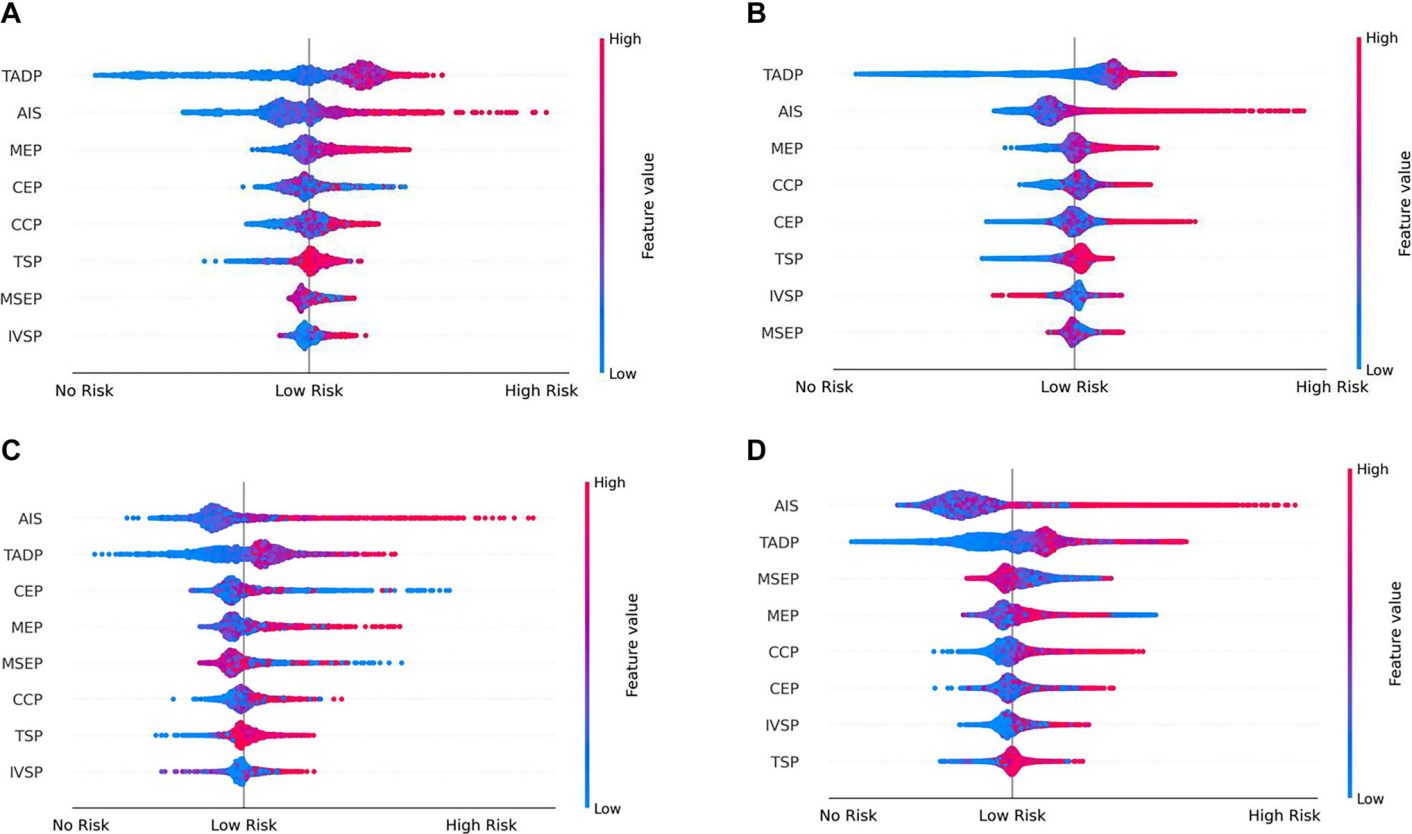
**The SHAP summary plots for the overspending forecasting [LightGBM (A and B) and RF (C and D)].** SHAP: SHapley Additive exPlanations; RF: Random forest. TADP: Total amount of discharged patients; AIS: Average inpatient stay; MEP: Medicine expenses percentage; CEP: Consumables expenses percentage; CCP: Critical cases percentage; TSP: Total surgery percentage, MSEP: Medical service expenses percentage; IVSP: IV-surgery percentage.

As shown in the SHAP summary plots (Figure 4), the vertical axis represents DPIs, while the right side of the horizontal axis indicates a positive correlation with high-risk overspending, and the left side indicates a negative correlation. The values of DPIs are presented in color: red indicates larger values, while blue indicates smaller values. Figure 4A and 4B shows that the top-ranking DPIs for high-risk overspending in the LightGBM model were TADP, AIS, MEP, and CEP. In these cases, higher values were positively associated with a greater risk of overspending. A similar DPI ranking for hospitals is shown in Figure 4. Combining the SHAP summary plots (Figure 4C and 4D) for RF, high-risk overspending was also positively correlated with TADP and AIS at both regional and hospital levels. However, Medical service expenses percentage (MSEP) had a greater impact at both levels.

### HIMS

**Figure 5.**
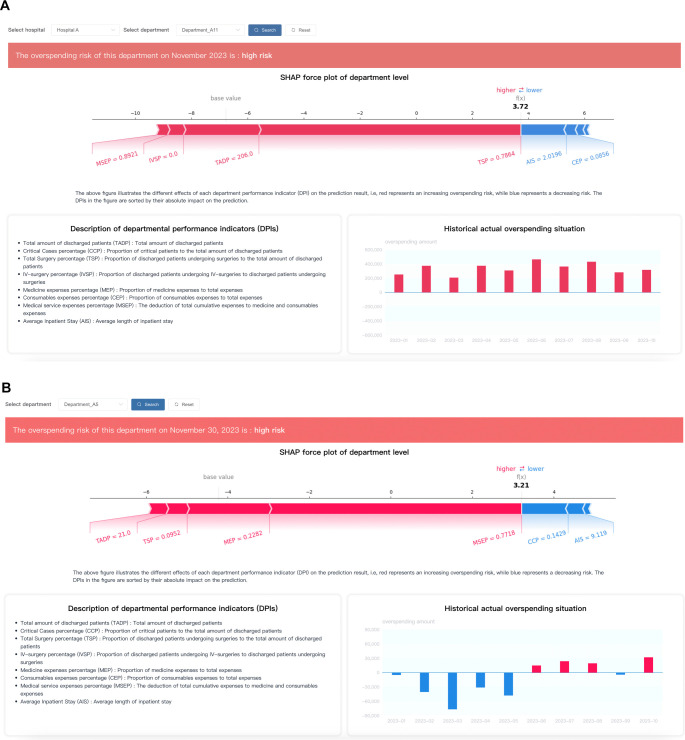
System user interface for regional (A) and hospital (B) administrators.

We have devised HIMS based on IORP, specifically designed for this purpose. Due to privacy concerns, we provide a demonstration of the system’s functionalities using partial test data in this context (http://prediction.overspending.risk.zxstech.com/). As shown in Figure 5A, regional administrators can select specific hospitals and their departments to access the latest monthly predictions of overspending risk. HIMS presents individual interpretative analyses, relevant DPI explanations (lower left), and historical overspending amounts (lower right). Daily overspending risk is also provided for hospital monitoring, allowing administrators to adjust budgets and prevent overspending in time (Figure 5B).

**Figure 6.**
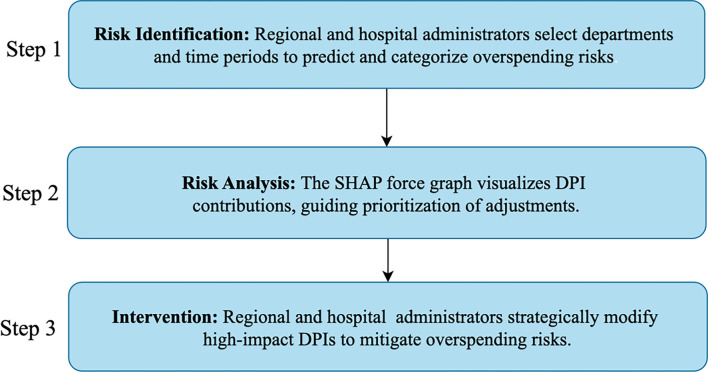
**Practical steps actionable by regional and hospital administrators**. SHAP: SHapley Additive exPlanations; DPI: Departmental performance indicators.

## Discussion

In the field of medical cost control, there has been significant concern about patient-level interventions for high expenditure [28–36]. By leveraging patient and departmental performance data, traditional statistical methods (e.g., linear regression and significance analysis) have identified factors related to high-expenditure departments, primarily attributing costs to complex patient cases, escalated drug expenditures, increased patient volume, inpatient services, and prolonged hospital stays [5, 9, 13, 37]. However, few studies have explored overspending risk factors for specific departments within regional and hospital contexts.

Our study validated some known factors associated with high health insurance expenditures and also identified additional factors—such as consumables expense percentage and total surgery percentage—that impact the balance of medical insurance expenditure. Based on these findings, our IORP modeling evaluated whether the 8 DPIs derived from patient medical records could help predict departmental overspending risks. To facilitate risk factor classification, SPC tools were applied during the modeling process. To the best of our knowledge, machine learning approaches have not previously been used for this specific purpose. In this study, we utilized four machine-learning algorithms (i.e., LR, RF, LightGBM, and ANN) to construct models for predicting regional- and hospital-level overspending. LightGBM achieved F1-scores of 0.78 and 0.82 for regional and hospital-level predictions, respectively, illustrating that medical records data contain valuable information for predicting departmental overspending status. Additionally, we developed an overspending risk system that provides risk predictions for regional and hospital administrators, with functionality for monitoring departmental health insurance overspending. Given the concerns of hospital departmental management, overspending on health insurance can adversely affect the quality of healthcare services. According to the IORP, drug and consumables expenses were identified as prominent predictors, suggesting that stricter control of these factors is needed in hospital management practices. AIS also emerged as a significant predictor of high-cost overspending, indicating that reducing patient hospital stays may help alleviate excessive spending. Several strategies can be implemented by hospital department managers. These include reducing postoperative infections, advancing medical technology to accelerate patient recovery, and improving hospital management protocols to reduce patient waiting times.

In addition, we found that TADP was a key determinant of high-risk overspending and proposed actionable interventions. These included optimizing bed scheduling, introducing nighttime procedures, and improving diagnostic appointment systems to relieve resource constraints. By implementing such measures, hospital administrators can proactively manage departmental expenditures, enhance financial oversight, and optimize resource allocation.

This work explores an improved prediction method for health insurance overspending risk through the use of hospital DPIs. Our proposed overspending risk prediction models and the integrated health information medical system demonstrate strong adaptability. The data types and formats input into the system are broadly applicable.

However, our study has several limitations. When applied to different hospitals or cities, the model’s hyperparameters may vary due to changes in population samples. Therefore, specific applications in each region may require appropriate adjustments and optimizations based on data characteristics and task requirements. Moreover, the DPIs used in our study reflect two primary aspects of healthcare administration: quality of care (TADP, CCP, TSP, IVSP, AIS) and operational efficiency (MEP, CEP, MSEP). In the future, our studies could further improve generalizability by incorporating socioeconomic variables (e.g., insurance coverage rates, rural/urban disparities) and automating DPI adjustments using federated learning techniques.

## Conclusion

In this paper, we developed IORP models utilizing DPIs tailored for regional and hospital administrators. Our process began with the collection of 549,910 discharged patient medical records from January 2022 to November 2023 in Wuxi, China. These records were organized into regional- and hospital-level departmental datasets, comprising 8,416 and 44,017 records, respectively. To analyze departmental overspending, we employed SPC to categorize the data into three risk groups: high risk, low risk, and no risk. Subsequently, we built regional- and hospital-level IORP models using machine learning methods including LR, LightGBM, RF, and ANN. Our experimental results indicated that the LightGBM algorithm demonstrated exceptional predictive capabilities, achieving an accuracy of 0.82 for both regional- and hospital-level models. To further enhance our analysis, we utilized SHAP to assess the importance of each DPI. This analysis highlighted four critical indicators strongly associated with departmental overspending: TADP, AIS, MEP, and CEP. Finally, we integrated the IORP models into the HIMS at the Affiliated Hospital of Jiangnan University. Using the steps outlined in Figure 6, administrators can monitor health insurance overspending. This integration significantly enhances departmental administrators’ ability to predict overspending risks, facilitating timely and accurate risk assessments. By optimizing departmental performance, the model supports the sustainable management of healthcare expenditures, ultimately contributing to improved financial health within healthcare institutions. As a result, the system proved instrumental in significantly reducing overall hospital expenses within just one year (2023–2024) under consistent departmental conditions: per capita medical costs decreased by 6.28%, per capita drug expenditures dropped by 12.18%, and per capita consumables costs were reduced by 14.1%. Through its application, the system has enabled regional and hospital departmental managers to optimize fiscal resources, resulting in enhanced financial management capabilities and more sustainable budgetary control across hospital departments.

## Supplemental data

Supplementary data are available at the following link: https://www.bjbms.org/ojs/index.php/bjbms/article/view/12051/3945.

## Data Availability

The datasets utilized and/or analyzed in this study can be obtained from the primary corresponding author upon reasonable request.
